# Mitochondrial TCA cycle metabolites control physiology and disease

**DOI:** 10.1038/s41467-019-13668-3

**Published:** 2020-01-03

**Authors:** Inmaculada Martínez-Reyes, Navdeep S. Chandel

**Affiliations:** 1 Department of Medicine, Northwestern University Feinberg School of Medicine, Chicago, IL USA; 2 Department of Biochemistry and Molecular Genetics, Northwestern University Feinberg School of Medicine, Chicago, IL USA

**Keywords:** Cell signalling, Metabolism

## Abstract

Mitochondria are signaling organelles that regulate a wide variety of cellular functions and can dictate cell fate. Multiple mechanisms contribute to communicate mitochondrial fitness to the rest of the cell. Recent evidence confers a new role for TCA cycle intermediates, generally thought to be important for biosynthetic purposes, as signaling molecules with functions controlling chromatin modifications, DNA methylation, the hypoxic response, and immunity. This review summarizes the mechanisms by which the abundance of different TCA cycle metabolites controls cellular function and fate in different contexts. We will focus on how these metabolites mediated signaling can affect physiology and disease.

## Abstract (web summary)

Mitochondrial metabolites contribute to more than biosynthesis, and it is clear that they influence multiple cellular functions in a variety of ways. Here, Martínez-Reyes and Chandel review key metabolites and describe their effects on processes involved in physiology and disease including chromatin dynamics, immunity, and hypoxia.

## Introduction

Mitochondria are cellular organelles that generate ATP and metabolites for survival and growth, respectively. Mitochondria are responsive to execution of commands from the nucleus. Gene expression changes from the nucleus promote mitochondrial biogenesis or increase mitochondrial respiratory activity to meet the cellular needs through a mechanism called “anterograde regulation”. However, it is clear now that mitochondria and the nucleus maintain a bidirectional regulation. Cells check whether mitochondrial metabolism is fit before they engage in complex and highly demanding cellular functions, including differentiation or adaptation to stress. Through a “retrograde signaling” mitochondria can regulate the expression of different genes to facilitate diverse cellular functions^1^. The mitochondrial requirements are diverse among different cell types and they are highly influenced by the microenvironment.

**Fig. 1:**
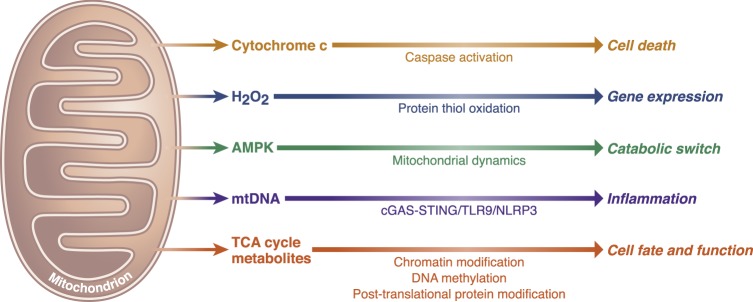
Essential signaling functions of mitochondria. Mitochondria have evolved distinct strategies to integrate environmental cues and communicate their fitness to the rest of the cell to maintain cellular homeostasis. Release of cytochrome c to invoke caspase-dependent cell death, release of reactive oxygen species to oxidize thiols within redox-regulated proteins, and induce gene expression and the activation of AMPK under energetic stress to control mitochondrial dynamics are three prominent mitochondrial-dependent signaling events. Additionally, release of mitochondrial DNA into the cytosol triggers inflammasome activation and pro-inflammatory responses through the cGAS–STING cytosolic DNA-sensing pathway. Signaling roles of TCA cycle metabolites are in part mediated by controlling chromatin modifications and DNA methylation, as well as post-translational protein modifications.

**Fig. 2:**
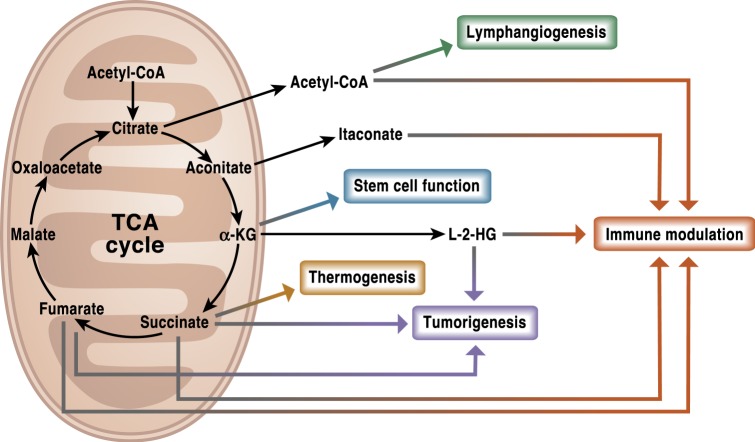
The TCA cycle is a signaling hub. TCA cycle metabolites have diverse non-metabolic signaling roles with important effects in physiology and disease. Metabolites such as acetyl-CoA, itaconate, succinate, fumarate, and L-2-HG can alter the response of both the innate and adaptive immune systems. Other functions like lymphangiogenesis or the maintenance of stem cells pluripotency have been associated with acetyl-CoA and α-KG, respectively. Succinate, L-2HG, and fumarate are well recognized oncometabolites that promote tumorigenesis. In addition to its intracellular functions, succinate can also act as a systemic signal to regulate thermogenesis upon exposure to cold temperature.

Four prominent mechanisms by which mitochondria communicate with the rest of the cell include the release of cytochrome c to induce cell death, activation of AMP-activated protein kinase (AMPK) to control mitochondrial fission and fusion, production of reactive oxygen species (ROS) to activate transcription factors, and the release of mitochondrial DNA (mtDNA) to activate immune responses^2–5^ (Fig. 1). Recent studies indicate a fifth mechanism whereby mitochondria release TCA (tricarboxylic acid) cycle metabolites to control cell fate and function (Fig. 2). TCA cycle metabolites were primarily considered as byproducts of cellular metabolism important for the biosynthesis of macromolecules such as nucleotides, lipids, and proteins. Although this is an essential function for the maintenance of cellular homeostasis, it is rapidly appreciated that metabolites in the TCA cycle are also involved in controlling chromatin modifications, DNA methylation, and post-translational modifications of proteins to alter their function. The purpose of this review is to highlight the signaling implications of TCA cycle metabolites and how changes in their abundance control physiology and disease.

## The TCA cycle and its regulation

### The TCA cycle overview

**Fig. 3:**
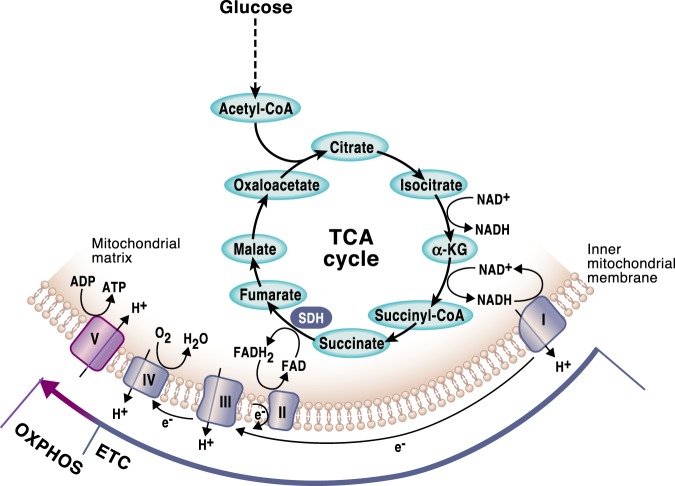
The TCA cycle and OXPHOS are tightly coordinated. In a series of enzymatic reactions the TCA cycle generates the reducing equivalents NADH and FADH2, which are required to transfer electrons to the mitochondrial respiratory chain, also known as the electron transport chain (ETC). As the electrons are funneled through the complexes in the inner mitochondrial membrane, a functional ETC generates a mitochondrial membrane potential that is used to produce ATP. This process requires the presence of oxygen and it is known as oxidative phosphorylation (OXPHOS). Mitochondrial complex I and II in the ETC replenish NAD + and FAD, respectively, allowing the oxidative TCA cycle to function. Succinate dehydrogenase is the only enzyme that participates in both the TCA cycle and the ETC.

The TCA cycle, also known as the citric acid cycle or the Krebs cycle, is a series of reactions in a closed loop that forms a metabolic engine within cells (Fig. 3). The TCA cycle constitutes an epicenter in cell metabolism because multiple substrates can feed into it. The TCA cycle begins with the reaction that combines the two-carbon acetyl-CoA, generated from fatty acids, amino acids or pyruvate oxidation, with a four-carbon oxaloacetate (OAA) to generate the six-carbon molecule citrate. In the second step, citrate is converted into its isomer, isocitrate. The cycle continues with two oxidative decarboxylation in which isocitrate is converted into the five-carbon α-ketoglutarate (α-KG) and subsequently into the four-carbon succinyl-CoA with the release of two molecules of CO_2_ and the generation of two NADH molecules. Next, succinyl-CoA converts into succinate, coupling it to the generation of GTP, which can be converted into ATP. Succinate is oxidized generating the four-carbon molecule fumarate. In this reaction, two hydrogen atoms are transferred to FAD, producing 2FADH. Importantly, the enzyme that carries out this step, succinate dehydrogenase (SDH), is also part of the electron transport chain (ETC) (Fig. 3). Next, fumarate gets converted into malate and further into OAA that combine with another acetyl-CoA molecule to continue the TCA cycle.

### The TCA cycle participates in both anabolism and catabolism

As the cycle runs, metabolites from the cycle are transported into the cytosol where they provide the building blocks for macromolecule synthesis^6^. For example, citrate is exported to the cytosol where it is converted to OAA and acetyl-CoA to promote nucleotide and lipid synthesis, respectively. Importantly, when TCA cycle intermediates are being shuttled away from the mitochondria for biosynthetic purposes, the cycle has to be replenished to keep it running. This process is referred to as anaplerosis. There are multiple inputs into the TCA cycle but two important anaplerotic mechanisms are the conversion of pyruvate to mitochondrial OAA by pyruvate decarboxylase and the activation of glutaminolysis, which converts glutamine to glutamate and subsequently to α-KG. The latter mechanism is often used when α-KG levels drop due to citrate being exported from the mitochondria into the cytosol for the de novo lipid synthesis. Interestingly, in cases where the ETC is impaired, the generation of some TCA cycle intermediates can be maintained by a process called glutamine-dependent reductive carboxylation^7^. In this pathway the cycle partially reverses itself to generate citrate from glutamine-derived α-KG by two subsequent reactions catalyzed by NADPH-dependent isocitrate dehydrogenase 2 (IDH2) and aconitase (ACO).

Enzymes of the TCA cycle evolved prior to the presence of oxygen on earth signifying the primary biosynthetic role of the TCA cycle. However, over time the TCA cycle has evolved into an important energy-producing pathway in eukaryotes. The primary function of one turn of the TCA cycle from an energy-generating perspective is to oxidize acetyl-CoA to two molecules of CO_2_. The completion of the TCA cycle generates ATP and the byproducts 3 NADH and 1 FADH2 that further feed the ETC complex I (NADH dehydrogenase) and complex II (SDH), respectively. Complexes I and II then pass their electrons through the ETC to ultimately produce ATP through oxidative phosphorylation (OXPHOS). The TCA cycle and OXPHOS are coupled since the oxidation of NADH and FADH2 in complexes I and II is required for the TCA cycle to keep functioning (Fig. 3).

### The TCA cycle is a tightly regulated pathway

The regulation of the TCA cycle and its constant feedback with OXPHOS is critical to keep the cells in a stable state. There are multiple positive and negative allosteric regulators that control the metabolic flux of the TCA cycle. NADH inhibits all the regulatory enzymes in the TCA cycle. Thus, in situations of ETC malfunctioning, NADH accumulates and the TCA cycle shuts down as a consequence. As NADH generates ATP through the ETC and OXPHOS, ATP is also an allosteric inhibitor of pyruvate dehydrogenase (PDH) and IDH. Thus, when cells have ample NADH and ATP, the cycle slows down. In contrast, high demands for ATP increases the ADP/ATP ratio and AMP levels, resulting in stimulation of the regulatory enzymes of the TCA cycle. Abundant acetyl-CoA inhibits PDH but activates pyruvate carboxylase to increase the formation of OAA from pyruvate and thus pair the levels of the two initiating metabolites of the cycle. Another intrinsic regulator is succinyl-CoA, which inhibits both citrate synthase and α-KG dehydrogenase to slow the cycle down. Likewise, an increase in OAA inhibits SDH and decelerates the cycle.

## The TCA cycle and cellular signaling

### Acetyl-CoA

Acetyl-CoA is a thioester between the two-carbon acetyl group (CH_3_CO) and a thiol, coenzyme A (CoA). As mentioned in the previous section, the maintenance of an acetyl-CoA pool is crucial to sustain the TCA cycle activity. To achieve this, acetyl-CoA can be generated from different sources and in multiple compartments. In mitochondria, acetyl-CoA can be generated from the oxidation of pyruvate, through fatty acid oxidation, by the degradation of the amino acids leucine, isoleucine, and tryptophan or through the mitochondrial enzyme aceyl-CoA synthetase short-chain family, member 1 (ACSS1)-mediated conversion of acetate. Acetyl-CoA can also be generated in the cytosol. Citrate can travel outside of the mitochondria through the dicarboxylate antiporter solute carrier family 25 (SLC25A1) and be converted back to acetyl-CoA and OAA by ATP citrate lyase (ACLY) both in the cytosol and in the nucleus. Finally, the cytosolic enzyme aceyl-CoA synthetase short-chain family, member 2 (ACSS2) can metabolize acetate into acetyl-CoA. Although this is not a common physiological pathway except in hepatocytes, cancer cells rely on this pathway to respond to the metabolic stress caused by limited nutrient availability^8,9^. The versatile participation of acetyl-CoA in multiple cellular processes makes it a critical metabolite to maintain cell homeostasis. Acetyl-CoA acts as a metabolic intermediate and as a precursor of anabolic reactions in the synthesis of fatty acids and steroids, and certain amino acids, including glutamate, proline, and arginine.

#### Acetyl-CoA regulates chromatin dynamics

**Fig. 4:**
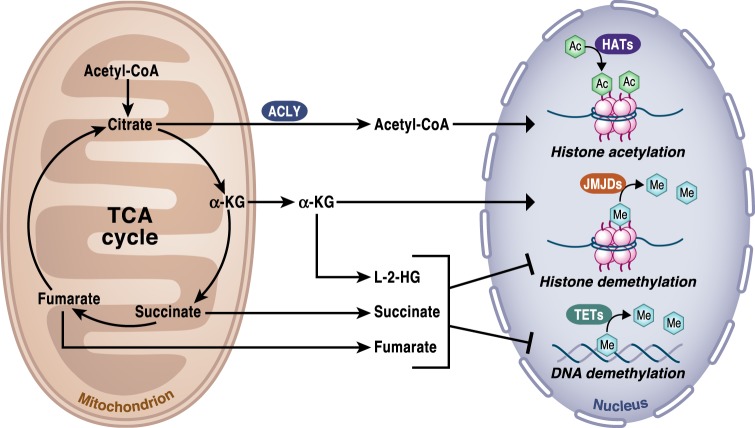
TCA cycle regulation of chromatin modifications and DNA methylation. The activity of the TCA cycle is essential to provide the metabolites that control chromatin modifications and DNA methylation. Specifically, histone acetylation by histone acetyltransferases (HATs) is dependent on the availability of acetyl-CoA, which provides the necessary acetyl groups to enable the reaction. Acetyl-CoA is produced in the cytosol by ACLY using citrate exported from the TCA cycle in mitochondria. α-ketoglutarate (α-KG) is an essential cofactor of 2-OGDD, including the histone demethylases JMJDs and TET DNA demethylases. Succinate is the product of 2-OGDD enzymes reactions and thus, when it accumulates, it works as an antagonist of the reaction. 2-HG and fumarate can also rewire the epigenetic landscape of the cells through inhibition of histone and DNA demethylases.

Likely the most prominent signaling function of acetyl-CoA is related to its ability to provide the acetyl groups for acetylation, one of the major post-translational protein modifications in the cell. Special attention has been paid to the contribution of acetyl-CoA as a necessary cofactor in the acetylation of histones, a mechanism known to alter the dynamics of chromatin to drive the epigenetic control of gene expression by activating transcriptional programs^10, 11^ (Fig. 4). Histone acetyltransferases (HATs) are the enzymes responsible for catalyzing the addition of acetyl groups in the histone N-terminal tails. The activity of HATs is sensitive to changes in the acetyl-CoA levels, which are in turn highly dependent on glucose availability, fatty acid oxidation and mitochondrial respiratory function^12–14^. Changes in acetyl-CoA abundance have been shown to affect global histone acetylation and gene expression^14, 15^. Importantly, genetic or pharmacologic disruption of ACLY activity can decrease histone acetylation^16^. Exogenous acetate can generate acetyl-CoA and maintain global histone acetylation when ACLY generated acetyl-CoA is limiting^17^.

In certain situations, specific genes are preferentially regulated upon changes in acetyl-CoA availability. For example, in glioblastoma multiforme (GBM) cell lines, limited glucose availability diminished acetyl-CoA levels and decreased the expression of genes involved in cell adhesion and migration by controlling the Ca^2+^-NFAT pathway^14, 18^. High levels of acetyl-CoA increased influx of calcium into the cells, activating and translocating NFAT to the nucleus. Once in the nucleus, NFAT recruits the lysine acetyltransferase (KAT) p300 to drive the site-specific regulation of H3K27ac and increased the expression of genes related to cell migration and adhesion to the extra-cellular matrix. The acetylation of this specific locus was maintained even in limited acetyl-CoA conditions when NFAT was constitutively active, which suggest that acetyl-CoA levels might not be rate limiting for site-specific histone acetylation. The results also suggest a dual regulation in the chromatin remodeling where histone acetylation can provide binding sites for regulatory proteins, but also specific transcription factors may be required for the acetylation of some histones marks in different contexts. Some transcription factors like Smad2 are regulated by acetylation, so the pool of acetyl-CoA in the cytosol and in the nucleus can also influence gene expression through this mechanism^19^.

#### Acetyl-CoA levels affect immune, cancer, and stem cells functions

Elevated histone acetylation as a consequence of high acetyl-CoA levels brings the cells to a pro-anabolic state increasing the expression of genes involved in cell growth and proliferation, including glycolytic enzymes^14^. Thus, cancer cells upregulate acetyl-CoA generating enzymes like ACLY to increase their proliferative capacity. Remarkably, inhibition of ACLY suppresses tumorigenesis^20^. The signaling pathways that lead to an increased acetylation of histones in high acetyl-CoA levels seem to vary in different contexts. For instance, recent findings in pancreatic cancer showed that high levels of acetyl-CoA and its use in the mevalonate pathway favored malignant progression of KRAS mutant acinar cells through the AKT-ACLY signaling axis^21^.

In other highly proliferative normal cells like T cells, increased cytosolic acetyl-CoA levels are required for histone acetylation to promote interferon-γ (IFNγ) production^22^. Along these lines, the production of acetyl-CoA by fatty acid β-oxidation regulates lymphangiogenesis through epigenetic changes regulated by the transcription factor PROX1^23^. Histone acetylation has also been linked to macrophages and dentritic cells (DCs) activation. Activated DCs and macrophages are characterized by a truncated TCA cycle that causes citrate levels to accumulate. As a consequence, lipopolysaccharide (LPS)-stimulated macrophages upregulate ACLY increasing cytosolic acetyl-CoA levels^24^. Upregulation of ACLY through NF-kB and STAT signaling pathways was shown to be necessary for the production of pro-inflammatory molecules, including nitric oxide (NO), ROS, and the prostaglandin E2 (PGE2) in induced macrophages.

Interestingly, the Akt-mTORC1 signaling axis has also been reported to regulate protein levels and activity of ACLY in macrophages activated by the polarizing anti-inflammatory signal IL-4^25^. An increased ACLY activity mediated histone acetylation and transcriptionally induced a subset of genes associated with cellular proliferation and production of chemokines^25^. These results indicate that a tight coordinated regulation and context-dependent metabolic control of histone acetylation is necessary to drive specific macrophages states. In embryonic stem cell differentiation, as the cells acquire a more quiescent phenotype the levels of acetyl-CoA and histone acetylation decrease^15^. Additionally, mitochondrial respiratory inhibition in hematopoietic stem cells decreases histone acetylation, which is associated with a block in cellular differentiation^26^. Overall, these studies highlight that acetyl-CoA is not just a passive acetyl group donor but rather an important signaling molecule involved in the regulation of the activity of specific transcription factors and histone marks that ultimately dictate cellular functions by regulating gene expression.

### α-ketoglutarate

α-KG, also known as 2-oxoglutarate, is an obligatory co-substrate for 2-oxoglutarate-dependent dioxygenases (2-OGDD), a large group of phylogenetically conserved enzymes, which catalyze hydroxylation reactions on various types of substrates, including proteins, nucleic acids, lipids, and metabolic intermediates producing CO_2_ and succinate. The activity of 2-OGDD is dependent on the intracellular ratio of α-KG to succinate or other inhibitors such as fumarate or 2-Hydroxyglutarate (2-HG). In addition to α-KG, these hydroxylation reactions require of Fe^2+^ as a cofactor and O_2_ as co-substrate. Ascorbic acid (vitamin C) also takes part in these reactions by inducing the reduction of oxidized Fe^3+^ to Fe^2+^ and restoring the activity of 2-OGDD enzymes. In humans, 2-OGDD play a key role in physiologically important processes such as responses to hypoxia and chromatin modifications. It is important to note that substrate intracellular concentrations including the levels of α-KG exceed the enzyme binding site affinity^27^. Thus, we propose that it is the accumulation of inhibitors of 2-OGDD reactions, i.e., succinate, fumarate or 2-HG above α-KG levels that modulate 2-OGDD enzymes to exert biological effects.

#### α-KG is a key modulator of the hypoxic response

**Fig. 5:**
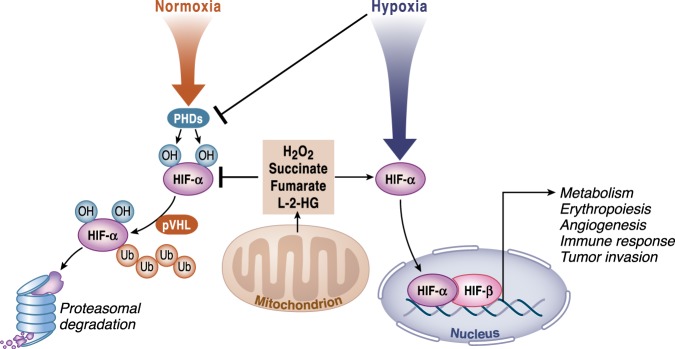
TCA cycle metabolites regulate HIF-α stabilization. Under normoxia, HIF-α is hydroxylated by prolyl-hydroxylases (PHDs) and targeted for proteasomal degradation by von Hippel-Lindau (pVHL) complex. Under hypoxic conditions, PHDs activity is inhibited preventing HIF-α hydroxylation, which causes its stabilization. HIF-α then translocates to the nucleus where it associates with HIF-1β to activate transcription of HIF target genes involved in metabolism, angiogenesis, erythropoiesis, immune responses, and tumor invasion. In normoxia, ROS released from mitochondria and accumulated levels of the metabolites succinate, fumarate and L-2-HG can inhibit the activity of PHDs causing a pseudohypoxia state.

Prolyl-hydroxylases PHD1–3 are 2-OGDD enzymes critical in the regulation of the transcription factor HIF-1, a master regulator of O_2_ homeostasis. In normoxia, proline residues located in the oxygen-dependent degradation domain of HIF-1α are hydroxylated by PHDs. The hydroxylation of HIF-1α is the signal for the recognition and poly-ubiquitination of HIF-1α by the VHL protein (von Hippel-Lindau tumor suppressor), which targets the protein for degradation in the proteasome. Under limited oxygen conditions or at reduced levels of α-KG or Fe^2+^, PHDs activity is impaired, resulting in HIF-1α or HIF-2α accumulation and translocation to the nucleus where it promotes changes in the expression of genes related to metabolism, erythropoiesis, and angiogenesis, as well as stem and immune cell function^28^ (Fig. 5). A combination of decreased oxygen levels and production of mitochondrial ROS diminishes PHDs to increase HIF-1α^29^. Mitochondrial ROS accumulation under normoxia can also inhibit PHDs to activate HIFs^30^. As discussed below, TCA cycle intermediates succinate, fumarate and L-2-HG under normoxic conditions can inhibit PHDs^31^. Interestingly, in the opposite direction, the reactivation of PHDs by increased intracellular α-KG levels in hypoxic cancer cells causes a severe metabolic impairment that lead to cell death^32^.

#### α-KG has multiple functions in physiology by regulating epigenetic changes

α-KG is also a required substrate of some chromatin-modifying enzymes, including the Jumonji C domain containing lysine demethylases (KDM2-7), which are the major histone demethylases and the ten-eleven translocation hydroxylases (TET1-3) involved in DNA demethylation (Fig. 4). Similar to PHDs, it has been recently reported that KDM6A and KDM5A activities are sensitive to changes in O_2_ levels^33, 34^. Histone methylation on single lysine (K) amino acid residues can activate or repress transcription depending on the particular residue and the level of methylation. DNA methylation modifies the chromatic structures and typically decreases gene expression by changing the structure of a single nucleotide. The availability of α-KG has a direct impact on gene expression and thus can modulate cellular fate decision by regulating histones and DNA demethylases. For example, high intracellular levels of α-KG (with the concomitant increase in the α-KG/succinate ratio) are necessary to maintain pluripotency by regulating multiple chromatin modifications^35^.

In macrophages, an important functional role has been attributed to α-KG in favoring an anti-inflammatory profile while repressing pro-inflammatory responses^36^. A primary reason for increased glutaminolysis in IL-4-activated macrophages is the increased production of α-KG that contributes to the acquisition of an anti-inflammatory phenotype by altering the activity of the histone demethylase Jmjd3. However, in classical macrophages activation following LPS stimulation, low levels of α-KG was found to dampen a pro-inflammatory response^36^. Mechanistically, α-KG suppresses IKKβ activation that is required for the pro-inflammatory effects driven by the nuclear factor-κB (NF-κB) pathway in a PHD activity-dependent manner. Furthermore, this same study described that α-KG produced from glutaminolysis promotes LPS-induced endotoxin tolerance^36^. These results highlight that targeting pathways involved in α-KG production offers attractive therapeutically opportunities in diseases associated with macrophage malfunction.

### 2-Hydroxyglutarate (2-HG)

#### 2-HG production and removal

Although 2-HG is not part of the TCA cycle, this α-KG derived metabolite can be generated by enzymes in mitochondrial matrix and cytosol. 2-HG competitively inhibits 2-OGDDs and exists in two isomers: L-2-HG and D-2-HG. Targets of both 2-HG include histone demethylases, TET enzymes that participate in DNA methylation and PHDs^37–39^. L- or D-2-HG abundance is limited in normal tissues however they can reach to millimolar concentrations under certain pathologic conditions^40, 41^. The isomer D-2-HG is accumulated as a consequence of gain-of-function mutations in the cytosolic and mitochondrial isoforms of IDH^42, 43^. Interestingly, *IDH1* and *IDH2* are the most frequently mutated metabolic genes in human cancer and are found in multiple tumor types, including gliomas, acute myelogenous leukemias (AMLs), and myelodysplastic syndromes (MDS)^42–45^. Single-point mutation of R132 in *IDH1* or R172 in *IDH2* reorganizes the active site, causing an increased affinity for NADPH to promote α-KG reduction at the expense of its principal substrate (isocitrate). The observation of D-2-HG accumulation in these tumors prompted the community to use the term oncometabolite for the first time. Currently, D-2-HG is being used as a biomarker to monitor the disease progression, and mutants IDH1/IDH2-specific inhibitors are in clinical trials for AML and glioma. Additionally, D-2-HG can be produced by the promiscuous activity of phosphoglycerate dehydrogenase (PHGDH), an enzyme frequently overexpressed in cancer. Normally, this enzyme catalyzes the first step in the de novo serine biosynthesis pathway where 3-phosphoglycerate (3PG) is converted to 3-phosphohydroxypyruvate (3PHP) coupled with NAD + reduction. However, in human breast cancer cell lines, PHDGH has been described to convert α-KG to D-2-HG in a NADH-dependent manner^46^.

**Fig. 6:**
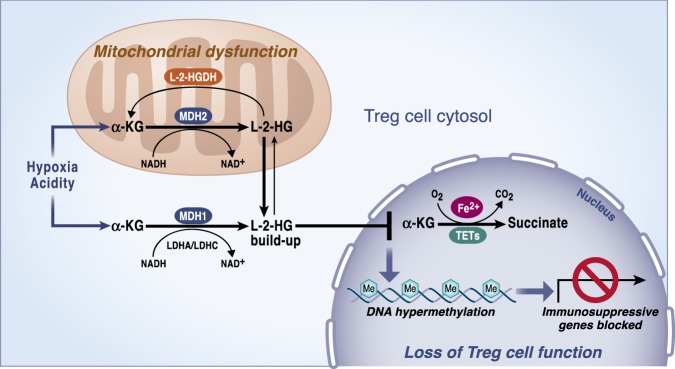
L-2-hydroxyglutarate (L-2-HG) regulates Treg cells function. Mitochondrial malate dehydrogenase (MDH2), its cytosolic counterpart (MDH1) and lactate dehydrogenases (LDH) A or C in the cytosol can exhibit enzyme promiscuity and catalyze α-KG reduction to L-2-HG. The reaction is coupled with NADH oxidation to NAD + . L-2-hydroxyglutarate dehydrogenase (L-2-HGDH) converts L-2-HG back to α-KG in mitochondria. Accumulated levels of L-2-HG inhibits the activity of TETs, which are enzymes involved in regulating DNA demethylation. TETs consume oxygen and α-KG as co-substrates producing CO_2_ and succinate. The reaction requires of Fe^2+^ as a cofactor. This mechanism has been observed to specifically repress immunosuppressive genes when mitochondrial complex III is impaired.

Malate dehydrogenases (MDH) 1 or 2 and lactate dehydrogenases (LDH) A or C can generate L-2-HG^47–49^ (Fig. 6). MDH2 and MDH1 normally catalyze the conversion of OAA into malate in mitochondria and cytosol, respectively. The conversion of α-KG to L-2-HG by MDHs is coupled with NADH oxidation to NAD + . In normal conditions, LDH catalyzes the interconversion of lactate and pyruvate. However, LDHA under hypoxia can generate L-2-HG. The ability of cells to increase L-2-HG in hypoxic conditions to regulate histone methylation levels, including H3K9me3 and to reduce cellular reductive stress by inhibiting key metabolic pathways indicates an important physiological role of L-2-HG^48, 50^. Acidic pH has also been described as a potent driver of L-2-HG production by favoring the promiscuous activity of LDHA and MDHs enzymes that utilize α-KG as an alternative substrate^51, 52^. Mechanistically, acidic pH generates a protonated form of α-KG that preferentially binds to LDHA^51^. An independent study showed that inhibition of enzymes involved in converting L-2-HG back to α-KG also accounts for the resulting L-2-HG accumulation observed in acidic microenvironments^52^. Importantly, both studies reported that accumulated levels of L-2-HG in acidic pH lead to stabilization of HIF-1α in normoxia. As α-KG availability directly influences the production of L-2-HG, these results bring the possibility of manipulating α-KG levels as a potential therapeutic strategy in acidosis.

Pathways for 2-HG removal are evolutionarily conserved. 2-HG can be converted back to α-KG through the action of the FAD-linked enzyme 2-hydroxyglutarate dehydrogenase (2-HGDH). Humans have two variants of this enzyme: D-2-hydroxyglutarate dehydrogenase (D-2-HGDH) and L-2-hydroxyglutarate dehydrogenase (L-2-HGDH), and both of them are located in the mitochondria. A deficiency in either of these two enzymes caused by germline transmission of homozygous mutations can lead to a disease known as 2-hydroxyglutaric acidurias (2-HGA). D-2-HGA is a rare disease, with symptoms, including macrocephaly, cardiomyopathy, mental retardation, hypotonia, and cortical blindness. L-2-HGA is a rare neurodegenerative disorder that causes hypotonia, tremors, epilepsy, mental retardation, and psychomotor regression. Notably, it has been reported that children with L-2-HGA developed medulloblastoma and glioblastoma multiforme, as well as Wilms tumor^53, 54^. Moreover, increased L-2-HG levels resulting from reduced expression of L-2-HGDH were observed in renal cancer, which suggest a potential tumorigenic effect for this isomer as well^55^.

#### 2-HG regulates immune and stem cells functions

All the promiscuous reactions leading to L-2-HG production share in common the oxidation of NADH, a driver of the reactions when it accumulates. Elevated NADH levels are a direct consequence of mitochondrial dysfunction, since one of the essential functions of complex I is NAD + recycling. Thus, inhibition of mitochondrial complex III in cancer cells has been shown to increase the production of 2-HG^56^. Likewise, disruption of complex III activity in hematopoietic stem cells (HSC) promoted an increase in the 2-HG levels^26^. Noteworthy, fetal HSC kept their proliferative capacity in respiration-deficient conditions but were unable to differentiate. More recently, it has been reported that loss of mitochondrial complex III in regulatory T cells (Tregs) resulted in a buildup of 2-HG^57^. Tregs are key mediators of immunological tolerance and homeostasis. In this study, mice lacking functional OXPHOS specifically in Tregs developed a lethal inflammatory disorder causing mice death between the third and the fourth week of life. Interestingly, complex III-deficient Tregs lost their suppressive capacity while their proliferation and survival remained unaffected. The transcriptomic profile of complex III-deficient Tregs compared to wild-type showed differences in the expression of immune regulatory molecules while maintaining stable FOXP3 expression, the master regulatory transcription factor of Tregs. In both contexts, in HSCs and Tregs, accumulated 2-HG levels seem to alter the cellular function, which is associated with hypermethylation of specific histone marks and DNA methylation.

Interestingly, in Tregs the loci of differentially down-regulated genes exhibited DNA methylation, suggesting a potential role for 2-HG in contributing to the autoimmune pathological phenotype of these mice. Accumulated 2-HG due to defective mitochondrial function then has the ability to mediate cellular signaling by directly and specifically impacting the expression of immunosuppressive genes to regulate Tregs without affecting other biological outcomes of this T cells subset (Fig. 6). This study adds more evidence on the interesting link between 2-HG levels and the regulation of cell fate in immune cells. For instance, D-2-HG has been shown to favor T helper 17 (T_H_17) cells differentiation, a T-cell population that promotes inflammation by increasing DNA methylation levels at the Foxp3 locus, which represses the differentiation of naïve CD4 + T cells towards induced Tregs^58^. Glutamate conversion to α-KG by the aspartate aminotransferase GOT1 was found to be essential for D-2-HG generation and thus for the regulation of T_H_17 and induced Tregs fate. Finally, in mouse CD8 + T cells, elevated levels of L-2-HG in response to T-cell receptor triggering have been reported to favor their differentiation and antitumor capacity^41^. Since the expression of 2-OGDDs likely is different among different tissues, the effects of 2-HG are likely to be context dependent. Collectively, 2-HG, a metabolite that gets accumulated through abnormal metabolic functions, exert most of the effects through non-metabolic mechanisms.

### Succinate

#### Succinate as an oncometabolite

Succinate is a TCA cycle metabolite that has multiple intracellular functions, as well as organismal functions. Succinate is considered an oncometabolite that accumulates due to inactivating mutations in SDH. SDH mutations are commonly found in several cancers, including hereditary paraganglioma (PGL) and pheochromocytoma (PCC)^59–62^. Succinate accumulation has an impact in gene expression regulation and promotes tumorigenesis through two main mechanisms. Succinate is the product of 2-OGDD enzymes reactions and thus its accumulation inhibits these enzymes. Consequently, changes in succinate have profound effects in histones and DNA methylation, changing the epigenetic landscape of the cells and gene expression (Fig. 4). In paraganglioma, mutations in the SDH genes were found to establish a DNA hypermethylator phenotype^63^. SDH-deficient cells displayed an increased 5-mC/5-hmC ratio and histone methylation, an effect that was reversed by exogenous addition of α-KG to the culture medium in vitro. DNA hypermethylation was associated with the silencing of key genes involved in neuroendocrine differentiation favoring malignancy. Further studies will determine if analogously to the effects of acetyl-CoA fluctuations in histone acetylation, particular programs get activated to promote histones or DNA methylation at specific loci instead of global changes in the presence of high succinate levels. Additionally, succinate can inhibit PHDs, promoting the accumulation of HIF-1α in the presence of oxygen, a phenomenon known as pseudohypoxia^64^.

#### Succinate has an important function in the regulation of innate immunity

The metabolic profile of LPS treated macrophages revealed a high abundance of succinate, which induces HIF-1α stabilization as well as the transcriptional activation of the pro-inflammatory cytokine IL-1β^65, 66^. Succinate oxidation by complex II is necessary to drive the production of IL-1β. Macrophages lacking the B subunit of SDH showed decreased IL-1β production and HIF-1α stabilization when stimulated with LPS. The inhibition of SDH in vivo diminished LPS-induced endotoxemia. LPS stimulation of both succinate oxidation to promote reduction of the ubiquinone pool accumulation and an increase in the mitochondrial membrane potential (ψm) raised the production of mitochondrial ROS through complex I-dependent reverse electron transfer (RET). The excess entry of electrons in the ubiquinone pool from complex II increases the propensity of electrons to go backward to complex I when the mitochondrial membrane potential is high. This triggers the generation of superoxide production from complex I. Thus, the addition of mitochondrial complex I or II inhibitors decrease IL-1β production and HIF-1α stabilization. The overexpression of an alternative oxidase (AOX), which is able to help ease up the excess of electrons in the ubiquinone pool, decreases LPS induction of ROS levels and production of IL-1β in macrophages. An independent study showed that *E. coli* or *S. enterica*-challenged macrophages stimulate mitochondrial complex II activity at the expense of complex I activity further supporting an importing role for SDH in promoting a pro-inflammatory phenotype^67^. The inhibition of complex II by treating mice with NPA, hampered the pro-inflammatory function of macrophages. Succinate-driven RET induction of ROS is not only limited to immune cells as ischemia reperfusion injury in heart and brain requires RET^68, 69^. Currently, strategies to diminish RET by using complex II or I inhibitors are being tested in the clinic.

#### Succinate has organismal effects

Beyond its intracellular signaling role, succinate release has been shown to signal through the G-protein-coupled receptor succinate receptor 1 (SUCNR1). The ligand for this receptor was unknown up until 2004 when He et al.^70^ described that binding of succinate to SUCNR1 induces an hypertensive effect by modulating the renin-angiotensin system. Since then, multiple signaling cascades have been described upon succinate binding to the receptor in various cell types, including dendritic cells and macrophages where it seems to contribute to their functionality in driving inflammation^71^. Taken together, these studies highlight that succinate oxidation is central and a main regulator of the immune effector cells functions. Finally, it is worth noting that recent findings describe succinate as a systemic molecule that activates thermogenesis in brown adipocytes upon cold exposure^72^. In this condition, brown adipocytes exhibited a high avidity to uptake elevated circulating succinate that was then oxidized by SDH increasing ROS levels and UCP1 activity. It will be interesting to see if additional TCA cycles metabolites can signal systemic responses in other contexts.

### Fumarate

#### Fumarate promotes tumor growth through diverse signaling functions

Accumulation of fumarate to millimolar levels due to inactivating mutations of fumarate hydratase (FH) is found in the genetic disorder fumaric aciduria, as well as the hereditary leiomyomatosis and renal cell cancer (HLRCC)^73^, in which fumarate causes hypermethylation of DNA by inhibiting TET enzymes to trigger epithelial-mesenchymal transition (EMT)^74^. Fumarate is a potent inhibitor of 2-OGDD enzymes^75, 76^ and thus, it also contributes to the pseudohypoxia state that characterized FH-deficient tumors by allowing HIF-1α stabilization even under normoxic conditions through the inhibition of the PHDs enzymes^77^. Fumarate is an electrophile and can cause succination of proteins, a process where fumarate binds and inactivates reactive thiol protein cysteine residues. This protein modification was first identified in the context of diabetes where elevated levels of fumarate contribute to a progressive deterioration of β cells function^78, 79^. Specifically, succination of critical cysteine in GAPDH, GMPR and PARK7/DJ-1 were found in mice lacking Fh1 that develop progressive glucose intolerance and diabetes at around week 6–8 after birth. In HLRCC, fumarate drives the succination of KEAP1, the negative regulator of the master antioxidant transcription factor NRF2^80–82^. Other studies have shown that high levels of fumarate increase ROS signaling by binding glutathione^83, 84^. Succination following fumarate accumulation has also been shown to cause the loss of the mitochondrial aconitase (ACO2) activity, which is crucial for iron–sulfur cluster binding^85^. Finally, the iron-responsive element binding protein 2 (IRP2) that promotes the transcription of transferrin and the family of iron–sulfur (Fe-SA) cluster biogenesis proteins have also been described to be succinated when fumarate accumulates in cancer cells^86^.

#### Fumarate is an anti-inflammatory signal

Fumarate also functions as an immunomodulator by controlling chromatin modifications (Fig. 4) and regulating protein succination. Specifically, the accumulation of fumarate through glutamine anaplerosis in response to pro-inflammatory insults has been shown to be necessary for trained immunity and inflammation by inhibiting KDM5 histone demethylase activity^87^. The inhibition of KDM5 increases the levels of H3K4me3, a marker of active gene transcription at the promoters of Tnfα and Il6 cytokines. Fumarate has also been shown to accumulate in LPS-activated macrophages^65, 88^. Furthermore, fumarate derivatives like dimethyl fumarate (DMF), a potent electrophile, can regulate T-cell functions^89^. DMF is currently being used in the clinic to treat autoimmune conditions, including multiple sclerosis (MS) and psoriasis. Using an unbiased proteomic approach, Blewett et al.^89^ discovered that different proteins in T cells are sensitive to covalent modifications of cysteines induced by DMF. Among these proteins, the authors identified protein kinase C θ (PKCθ) that can no longer associate with the costimulatory receptor CD28 in the presence of DMF preventing T-cell activation. Importantly, the authors showed how a mutation of these DMF-sensitive cysteines impaired PKCθ-CD28 interactions and T-cell activation recapitulating the effects observed with DMF^89^. Recently, succination of GAPDH that leads to the inactivation of its enzymatic activity has also been identified in peripheral blood mononuclear cells (PBMCs) from mice and patients with MS treated with DMF^90^. Endogenous fumarate was also found to inhibit GAPDH through succination in mouse and human macrophages. These results suggest that DMF modulate the activity of immune cells by negatively regulating glycolysis, a metabolic pathway required for the activation and proliferation of different subsets of T cells^91^.

### Itaconate

#### Itaconate is an important immunomodulator and antibacterial agent

Itaconate is derived from the decarboxylation of the TCA cycle intermediate cis-aconitate. Accumulation of itaconate occurs in the lungs of mice infected with *Mycobacterium tuberculosis*^92^ and in macrophages activated with LPS^93^. The immune-responsive gene 1 protein (IRG1) is the enzyme responsible for itaconate production. IRG1 was consequently renamed as cis-aconitate decarboxylase after this discovery. IRG1 loss of function in macrophages infected with *Salmonella enterica* had reduced aconitate levels and decreased anti-bactericidal activity^94^. The anti-bactericidal properties of itaconate are derived from its ability to inhibit isocitrate lyase, a key enzyme of the glyoxylate shunt, a pathway required for the survival of many parasites especially under poor glucose conditions. LPS also induces IRG1 to trigger the accumulation of itaconate, which consequently limits IL-1β production^95, 96^.

#### Itaconate induces electrophilic stress

Initially, itaconate was shown to inhibit SDH thus limiting succinate oxidation and this was assumed to be the primary mechanism by which itaconate limits inflammation^95, 96^. However, itaconate is a relatively weak competitive inhibitor of SDH raising the possibility that there are other mechanisms to account for itaconate’s anti-inflammatory activity. Recent studies indicate that itaconate activates pathways downstream of the antioxidant transcription factor NRF2, as well as NRF2 independent mechanisms that contribute to its anti-inflammatory properties in activated macrophages. Itaconate has electrophilic properties that can disrupt KEAP1 interaction with NRF2^97^. Itaconate can also inhibit the NF-κB inhibitor IκBζ to dampen LPS stimulation of inflammation^98^. Interestingly, derivatives of itaconate such as octyl-itaconate or dimethyl-itaconate, which are strong electrophiles, diminish pro-inflammatory cytokine production in vitro and in vivo^97, 98^. The administration of dimethyl-itaconate in vivo ameliorated the outcome of an IL-17–IκBζ-driven skin pathology in a mouse model of psoriasis. These results significantly extended the knowledge of itaconate biology and raised the possibility of using itaconate derivatives as a therapeutic agent in autoimmune diseases. However, since high levels of itaconate might cause a B12 deficiency, the safety of long-term exposures to itaconate needs to be evaluated^99^.

## Conclusion

In the past two decades, mitochondrial biology has undergone a renaissance partly due to the appreciation that mitochondria have important biological functions beyond ATP and macromolecules production. Indeed, mitochondria have evolved from passive to active players in determining cell fate and function. Mechanistically, TCA cycle metabolites have been demonstrated to control transcription factors and chromatin modifications to change cell function and fate. However, in many contexts the molecular details of how changes in TCA cycle metabolites abundance affect the expression of specific genes remains to be elucidated. Future investigations might also discover additional mechanisms by which TCA cycle metabolites exert signaling functions beyond post-translational modifications. Emerging evidence indicates that beyond cell autonomous functions, TCA cycle metabolites control physiology through non-cell autonomous functions. Discovering systemic effects of metabolites and their role in communicating different parts of the body will be of much interest for the field in the upcoming years. A fascinating development is the use of derivatives of TCA cycle metabolites to ameliorate inflammatory diseases in humans. We hope to see more of the recent findings of TCA cycle signaling effects being translated into the clinic. Going forward, we predict that TCA cycle metabolites will continue to shed new light on biology, physiology, and diseases.
